# Noncanonical Roles of h*α*-syn (A53T) in the Pathogenesis of Parkinson's Disease: Synaptic Pathology and Neuronal Aging

**DOI:** 10.1155/2020/6283754

**Published:** 2020-03-21

**Authors:** Qing-Jun Wang, An-Di Chen, Hai-Chao Chen, Dong-Xin Wang, Yi-Ting Cai, Jie Yin, Yu-Hong Jing, Li-Ping Gao

**Affiliations:** ^1^Institute of Anatomy and Histology & Embryology, Neuroscience, School of Basic Medical Sciences, Lanzhou University, Lanzhou, China; ^2^Institute of Biochemistry and Molecular Biology, School of Basic Medical Sciences, Lanzhou University, Lanzhou, China; ^3^Key Laboratory of Preclinical Study for New Drugs of Gansu Province, School of Basic Medical Sciences, Lanzhou University, Lanzhou, China

## Abstract

The motor and nonmotor symptoms of PD involve several brain regions. However, whether *α*-syn pathology originating from the SNc can directly lead to the pathological changes in distant cerebral regions and induce PD-related symptoms remains unclear. Here, AAV9-synapsin-mCherry-human SNCA (A53T) was injected into the unilateral SNc of mice. Motor function and olfactory sensitivity were evaluated. Our results showed that AAV9-synapsin-mCherry-human SNCA was continuously expressed in SNc. The animals showed mild motor and olfactory dysfunction at 7 months after viral injection. The pathology in SNc was characterized by the loss of dopaminergic neurons accompanied by ER stress. In the striatum, h*α*-syn expression was high, CaMK*β*-2 and NR2B expression decreased, and active synapses reduced. In the olfactory bulb, h*α*-syn expression was high, and aging cells in the mitral layer increased. The results suggested that h*α*-syn was transported in the striatum and OB along the nerve fibers that originated from the SNc and induced pathological changes in the distant cerebral regions, which contributed to the motor and nonmotor symptoms of PD.

## 1. Introduction

The typical pathology of Parkinson's disease (PD) is characterized by the aberrant conformation of *α*-syn and the formation of Lewy bodies (LBs) [1–3]. Monomer *α*-syn is soluble and unfolded under physiological condition. *α*-syn is prone to form an *α*-helix structure when its N-terminal binds to the phospholipids of cellular membrane [4]. Under pathological conditions, *α*-syn initially forms soluble oligomers, also called profibrils, which are spherical and annular molecules with linear morphology under the electron microscope [5]. Gradually, profibrils become insoluble and combine with each other to form fibers [6]. A53T and A30P mutations of *α*-syn have a higher probability of aggregating to form fibrils than wild type [7]. *α*-syn fibers can also be deposited in nerve cells to form inclusion bodies known as LBs (in the cell body) and Lewy neurites (LNs, in the terminals of neurons) [6]. In the past half century, LBs have been gradually used as a neuropathological marker for PD patients [8–10]. *α*-syn could have formed into LBs and LNs, damaging the neurons and synapses and causing motor and nonmotor dysfunctions [11–13].

LBs are gradually distributed in various brain regions during the development of PD. LBs first appear in the olfactory bulb, substantia nigra (SNc), cortex, and other brain regions [14, 15]. Braak and Del Tredici found that LBs formed by *α*-syn were regularly distributed in the brain of sporadic PD patients. In the early stage, LBs first appeared in the dorsal nucleus of the vagus nerve and olfactory bulb, then in the basal nuclei and midbrain, and finally in a wide area of the neocortex [16]. The distribution of LBs in the brain region was consistent with the diversity of clinical symptoms in PD patients. These patients first showed nonmotor symptoms, such as olfactory and sleep disorders, and subsequently exhibited motor symptoms at the later stage [17–19]. Although Braak found that the pathology of LBs gradually appeared from one brain region to another in PD patients, the frequent appearance of *α*-syn in specific brain areas, such as the SNc, remains unclear. In recent years, studies have shown that the progress of the pathology of LBs is similar to the transmission of the prion virus, that is, *α*-syn misfolded protein can be transferred from one brain region to another and then deposited [20–22]. This condition is supported by the treatment of PD patients with embryonic neuron transplantation, in which LBs are found in the transplanted embryonic neurons. Four separate autopsies have reported the presence of LBs with *α*-syn as the main component and deposed in dopaminergic neurons at 10 to 22 years after transplantation [23–26].

Although LBs or LNs with *α*-syn as the main component can be widely distributed in multiple brain regions, the sensitivity to neuronal damage appears to be mainly focused in dopaminergic neurons in the SNc [27, 28]. To date, the mechanisms implicated in the selective neural degeneration remain unclear. The etiopathology of their death remains unknown although many varied and frequent interacting mechanisms, including mitochondrial dysfunction, protein mishandling, inflammation, oxidative stress, genetic and environmental factors, and normal aging, have been proposed [29–32]. These factors are not necessarily confined to SNc dopamine neurons but include other classes of neurons in other brain regions. The initial risk factors from the SNc or other brain regions cannot be distinguished based in h*α*-syn (A53T) transgenic mice. Therefore, in this study, AAV9-synapsin-human SNCA (A53T) virus was injected in the right side of SNc. The pathological changes in the right olfactory bulb and right striatum were observed at 7 months after injection.

Dopamine neurons in SNc projected to the striatum and formed synaptic connections. Whether the continuous expression of h*α*-syn in dopamine neurons of SNc affected synaptic function in the striatum, so the expression of CaMK*β*-2 and NR2B were evaluated, in which CaMK*β*-2 is related to the release of presynaptic vesicles [33], and NR2B is related to the function of NMDA receptor in postsynaptic membrane [34], while CREB is a molecule closely related to synaptic plasticity [35]. Considering the function of dopamine projection in the striatum is related to the distribution of dopamine receptors, the levels of D1R and D2R in the striatum were measured. Additionally, the synaptic structural changes in the striatum were analyzed using electron microscopy combined with semiquantitative technique, to evaluate the effect of continuous high expression of h*α*-syn on synaptic plasticity in the striatum from both functional and structural aspects. GRP78 and CHOP are important regulators in ER stress induced by protein accumulation [36]. This study is aimed at revealing the influence of h*α*-syn (A53T) in the SNc on distant brain regions, such as striatum and olfactory bulb, its pathological mechanism, and whether the high expression of h*α*-syn (A53T) in the SNc can directly cause motor dysfunction and impairment of olfactory sensitivity.

## 2. Materials and Methods

### 2.1. Animals

Male C57BL/6 mice weighing approximately 20–22 g were purchased from the Experimental Animal Center of Lanzhou University. All animals were raised under standard conditions at 22°C to 25°C, 40%–60% humidity, and 12 hours light cycle with feeding and drinking ad libitum. The mice were divided into experimental and control groups. The experimental mice were injected with AAV9-synapsin-human SNCA (A53T) in the right SNc, and the control mice were injected with AAV9-synapsin-mCherry in the right SNc. All animal experiment protocols were approved by the Animal Ethics Committee of Lanzhou University.

### 2.2. Reagents

Isoflurane was obtained from RWD Life Science Co. (Shenzhen, China). AAV9-synapsin-human SNCA (A53T) and AAV9-synapsin-mCherry were developed by Ruizhen Biotechnology Co. (Shandong, China). Rabbit polyclonal anti-Grp78, anti-TH, and anti-p62 antibodies were purchased from Abcam (Cambridge, UK). Rabbit polyclonal anti-CHOP, anti-*β*-camkII, and anti-NR2B antibodies were purchased from Bioworld Technology Co., Ltd. (Nanjing, China). Mouse monoclonal anti-GAPDH antibody was purchased from Immunoway Biotechnology Company (Texas, USA). PVDF membrane and ECL agent were obtained from Millipore (Bellerica, MA, USA). RNAiso Plus, cDNA reverse transcription kit, and real-time PCR (RT-PCR) mix were purchased from TAKARA Biotechnology Co., Ltd. (Dalian, China).

### 2.3. Stereotactic Injection

Stereotactic viral injections were performed under general isoflurane anesthesia by using a stereotactic instrument. AAV9-synapsin-mCherry-human SNCA (A53T) or AAV9-synapsin-mCherry (500 nl) was injected in the right SNc (coordination: AP, −3.2 mm; ML, 1.2 mm; DV, 4.3 mm) at a slow rate (125 nl/min) by using a syringe pump. The injection needle was withdrawn 5 min after the end of infusion. The accurate location of injection sites and viral infectivity were confirmed in all mice post hoc by preparing the sections.

### 2.4. Behavior Test

#### 2.4.1. Open Field Test (OFT)

Open field test (OFT) was performed by using an OFT-100 instrument (Taimen, Chengdu, China). The size of the experimental box was 50 cm × 50 cm × 40 cm. During the experiment, the mice were placed in the experimental box, and the freezing time, movement distance, and residence time in the middle area of the box were recorded for 5 min.

#### 2.4.2. Cylinder Test

Forelimb asymmetry in spontaneous movement was evaluated by using the cylinder test based on the previous description. The animal was placed in a transparent cylinder, and its progress was recorded for 10 min. Two mirrors were placed behind the cylinder to facilitate recording of all forelimb touches. The forelimb touch times were recorded and counted in 10 min, and the ratio was calculated as follows: ratio = right forelimb touch times/left forelimb touch times.

#### 2.4.3. Pole Descent

A 0.5 m long pole with a diameter of 1 cm and wrapped with nonadhesive shelf liner was placed in the home cage to facilitate the grip of animals. We followed the methods of Tim Arentsen et al. [37]. In brief, animals received two days of training to descend from the top of the pole and into the home cage. The animals were given three trials on the first day of training. They were placed with their heads down 1/3 of the distance above the floor on the first trial, 2/3 of the distance on the second trial, and placed at the top on the third trial. On the second day of training, the animals were given three trials to descend with their heads down from the top of the pole. On the test day, the animals were placed with their heads down on the top of the pole and timed on their way back into the home cage. Timing began when the experimenter released the animal and ended when one hind limb reached the home cage base.

#### 2.4.4. Olfactory Test

Olfactory preference test was performed based on the previous description and modified. The bottom of the test box (25 cm × 15 cm × 10 cm size) was covered with wood chips. The mice fasted for 24 hours before the experiment. Olfactory habituation training was conducted as follows: 100 *μ*l peanut butter (0.6 g dissolved in 1 ml distilled water) was set on a 5 cm × 5 cm filter paper, which was placed on one side of the test box. The mice were returned in the original cage after their stay in the test box for 15 min. After 5 min, the mice were placed in the test box again and stayed for 15 min. After 30 min, the olfactory test was carried out. First, the filter paper with 100 *μ*l distilled water was buried under the wood chips, and the length of time until the mice found the filter paper was recorded as control. Second, the filter paper with 100 *μ*l peanut butter was replaced and buried under the wood chips, and the length of time until the mice detected the filter paper was recorded.

#### 2.4.5. Food Burial Experiment

The food burial test was preformed based on the previous description. The bottom of the test box (25 cm × 15 cm × 10 cm size) was covered with wood chips, and 15 pieces of chow food were buried in the wood chips. After fasting for 24 hours, the mice were placed in the test box, and the length of time the mice found all the chow food was recorded.

#### 2.4.6. Three-Chambered Social Approach Task

Sociability and social cognition were evaluated in a three-chambered apparatus, as previously described. A rectangular three-chambered box made of clear plexiglass was divided into three equal-sized chambers (45 cm × 20 cm × 35 cm). The walls dividing the chambers had a small rectangular opening (10 cm × 6.5 cm), providing access to the adjacent chambers. Clear rectangular plexiglass doors were used to close the openings as needed. The test session began with a short habituation (i.e., 1 min) at the center chamber without access to either of the side chambers, followed by a 10 min habituation session in the entire empty box with access to all three chambers. The test mouse was briefly confined in the center chamber as the experimenter placed the stimuli. To evaluate the social approach behaviors in a live unfamiliar mouse (with the same weight, sex, and strain as the test mouse), a novel C57BL/6 mouse previously habituated to a grid enclosure (inverted wire pencil cup) was placed in one of the side chambers. A control novel object, an identical empty grid enclosure devoid of social odors, was placed in the other side chamber. The location of the novel object and the novel mouse alternated between the left and the right sides chambers across subjects. After the objects were positioned in the side chambers, the two doorways were simultaneously opened, and the test mouse was allowed access to all three chambers for 10 min. After 10 min of testing, the test mouse was shortly confined in the center chamber again. To assess social cognition, a novel C57BL/6 stimulus mouse was placed in the chamber that previously contained the empty grid enclosure. With the stimuli mice positioned in their respective enclosures, the doorways were opened, and the test mouse freely explored all three chambers for 10 min. The time spent in each chamber and time spent around the enclosures (near the novel object, novel mouse, or familiar mouse) was recorded by a video camera positioned directly above the testing apparatus and analyzed by an automated tracking system.

### 2.5. Preparation of Brain Tissue

At 15 days and 3 months after the viral injection, three mice were randomly selected from the experimental and control groups, and viral infectivity and h*α*-syn expression were determined. At 7 months after injection, the behavior was first measured, and the mice in each group were randomly divided into three parts, namely, animals (*n* = 8) that will be used to prepare the fresh tissue of the specific brain region, animals (*n* = 8) that will be used to prepare the brain section, and animals (*n* = 3) that will be used for electron microscopic examination.

#### 2.5.1. Preparation of Fresh Brain Tissue

The mice were subjected to an intraperitoneal local anesthetic of 3% pentobarbital sodium (45 mg/kg). Their brains were removed, and the right midbrain and striatum were separated under stereomicroscope, frozen with liquid nitrogen, and stored in the refrigerator at −80°C before use.

#### 2.5.2. Preparation of Brain Section

The mice were subjected to an intraperitoneal local anesthetic of 3% pentobarbital sodium (45 mg/kg). Their thoracic cavity was opened, and 0.9% normal saline was perfused at a rate of 30 rpm/min for 3 min in the left ventricular, then with 4% paraformaldehyde at a rate of 30 rpm/min for 2 min, and then at a rate of 18 rpm/min for 10 min. The entire brain was fixed with 4% paraformaldehyde overnight and placed in 20% and 30% sucrose until they sank to the bottom of the container. The cryostat serial section with 25 *μ*m thickness was made by using a cryostat microtome.

### 2.6. Immunofluorescence

Fifteen days after viral injection, the brain sections of SNc were selected and incubated with polyclonal rabbit anti-TH antibody (1 : 200) at 37°C for 1 h and at 4°C overnight. The sections were rinsed with 0.01 M phosphate-buffered saline (PBS) and incubated with a second antibody (1 : 100) conjugated with FITC at 37°C for 1 h. The sections were rinsed with 0.01 M PBS and stained with DAPI. The sections were observed by using a fluorescence microscope, and the number of TH-labeled neurons, mCherry-positive neurons, and TH and mCherry colabeled neurons was counted in the SNc. Briefly, according to the mouse brain atlas of George paxinos, three sections were selected: Bregma-2.7, Bregma-2.95, and Bregma-3.2, interval 250 *μ*m. The number of TH-positive cells in each section was counted, and the average value of the three sections represented the number of TH-positive cells in the SNc of one mouse.

### 2.7. Immunohistochemistry

SNc, striatum, and olfactory bulb sections were selected and quenched in 0.3% hydrogen peroxide (H_2_O_2_) for 20 min to remove endogenous peroxidase activity. After several washings with 0.01 M PBS, the sections were incubated in PBS containing mouse anti-*α*-syn (A53T) antibody (1 : 200), 0.3% Triton X, and 0.1% goat serum for 24 h at 4°C. The incubated sections were washed and incubated for 90 min in biotinylated goat antirabbit antiserum (1 : 200), followed by washing with 0.01 M PBS. Then, the sections were incubated with strep-avidin–biotin peroxidase complex (1 : 200) for 90 min and immersed in 0.02% 3,3-diaminobenzidine containing 0.01% H_2_O_2_ in 0.01 M PBS for development.

### 2.8. Electron Microscopy

Glutaraldehyde (3% in PBS)-fixed dorsal striatum and SNc was postfixed with 1% OsO4 for 90 min and dehydrated with graded ethanol. The samples were then embedded with Epon 812 and polymerized at 72°C for 48 h. Four ultrathin sections (50–70 nm) were selected at every 1.0 *μ*m interval per mouse, stained with lead acetate and uranyl, and observed with a JEM1230 microscope (Tokyo, Japan). The images were captured with the Gatan image system (NY, USA).

### 2.9. Western Blot

Four mice were randomly selected from each group, anesthetized with pentobarbital, and the right midbrain and right striatum were obtained and placed in ice-cold PBS. The samples were frozen in liquid nitrogen and homogenized, and the protein fractions were prepared by using an extraction reagent containing protease inhibitors. The total proteins (30 *μ*g) were fractionated in 10% SDS–PAGE and transferred to PVDF membranes. The membranes were blotted with anti-p62 (1 : 1000), anti-Grp78 (1 : 1000), anti-CHOP (1 : 1000), anti-CaMk*β*-2 (1 : 1000), anti-NR2B (1 : 1000), and anti-GAPDH (1 : 5000) antibodies at 4°C overnight. After washing with 0.01 M TBST, the membranes were blotted with horseradish peroxidase conjugated secondary antibody (1: 5000), and the immunoreactive protein bands were visualized by using enhanced chemiluminescence.

### 2.10. Real-Time PCR

Total RNA was extracted from the right striatum by using an RNAiso Plus reagent (Takara Biotech, Co., Ltd., Dalian, China) in accordance with the manufacturer's instructions. DNA contamination was removed with RNase-free DNase. cDNA was synthesized from 1 *μ*g of RNA with M-MuLV reverse transcriptase according to the manufacturer's instructions. Quantitative RT-PCR was performed by using a PIKoREAl96 detector (Thermo Scientific, USA). The mRNA levels of DR1, DR2, and CREB in triplicate samples of reverse-transcribed cDNA were evaluated with a SYBR Green PCR Master Kit (Promega Corporation, USA) in accordance with the manufacturer's instructions. The primers for mouse CREB were 5′-GACAACCAGCAGCAGAGTGGAGATG-3′ (forward) and 5′-TGGATACCTGGGCTAATGTGG-3′ (reverse). The primers for mouse DR1 were 5′-GGTGGAGGAGGACTGGTGTCAA-3′ (forward) and 5′-CTTGGAAATCACTTTGCCTGGA-3′ (reverse). The primers for mouse DR2 were 5′-CCTTCATCGTCACCCTGCTGG-3′ (forward) and 5′-CTCCATTTCCAGCTC CTGAG-3′ (reverse). The mouse GAPDH primers were 5′-GCGAGACCCCACTAACATCAA-3′ (forward) and 5′-GTGGTTCACACCCATCACAAA-3′ (reverse). The assays were initiated for 5 min at 95°C, 40 cycles of 15 s at 94°C, and 1 min at 60°C. The threshold cycles of the target gene and GAPDH were calculated. The amplification of DR1, DR2, and CREB cDNA was normalized to the expression of GAPDH. The relative mRNA expression levels of CREB, DR1, and DR2 were calculated by using a 2^-*ΔΔ*CT^ method.

### 2.11. *β*-Gal Staining

Olfactory bulb sections were selected and rinsed with 0.01 M PBS and fixed with 1% paraformaldehyde at room temperature for 30 min. The sections were rinsed with 0.01 M PBS, stained with the *β*-gal kit according to the operation manual, and counterstained with 1% neutral red for 5 min. Finally, the sections were dehydrated and observed.

### 2.12. Statistical Analysis

Data were expressed as mean ± SEM. One-way ANOVA was conducted for multiple group comparisons, and Tukey's post hoc analysis was used to evaluate the significance of the paired groups. In all analyses, *p* < 0.05 was considered significant.

## 3. Results

### 3.1. High Expression of h*α*-syn in the SNc

**Figure 1:**
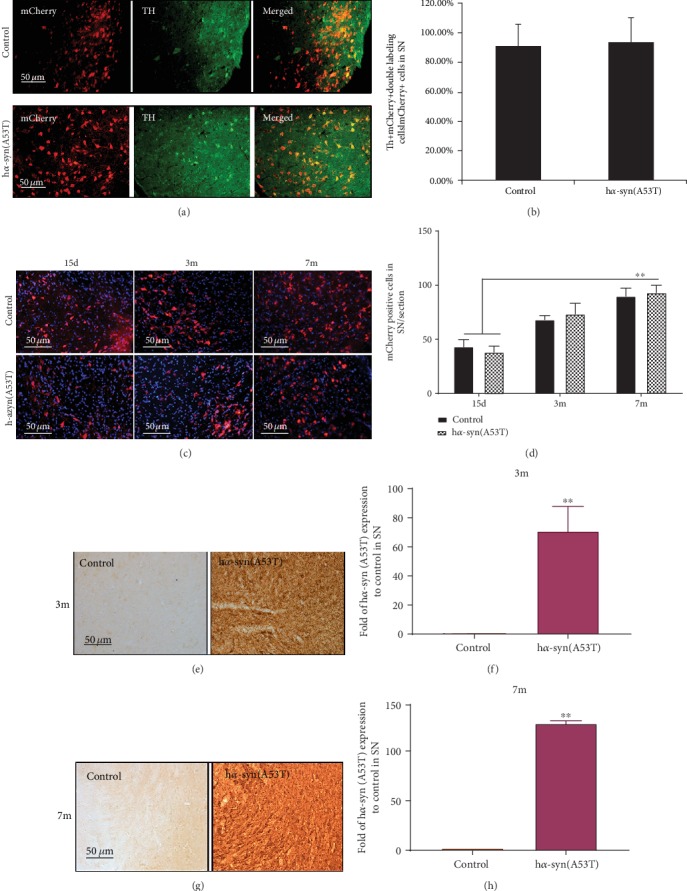
Exogenous high h*α*-syn expression in the SNc. (a) Representative images showing mCherry-positive, TH-positive, and colabeled cells at 15 days after viral injection. (b) Ratios of colabeled cells in the total mCherry-positive cells in the SNc (*n* = 3). (c) Representative images showing mCherry-positive cells in the SNc at 15 days, 3 months, and 7 months after viral injection. (d) Number of mCherry-positive cells in the SNc at 15 days, 3 months, and 7 months after viral injection (*n* = 3). (e) Representative images showing h*α*-syn expression in the SNc by using immunohistochemistry at 3 months after viral injection. (f) Quantitative analysis of h*α*-syn expression in the SNc at 3 months after viral injection (*n* = 3). (g) Representative images showing h*α*-syn expression in SNc by using immunohistochemistry at 7 months after viral injection. (h) Quantitative analysis of h*α*-syn expression in the SNc at 7 months after viral injection (*n* = 5). ^∗∗^*p* < 0.01 compared with the control group.

At 15 days, 3 months, and 7 months after viral injection, mCherry-positive cells in ipsilateral SNc were observed by using a fluorescence microscope, and h*α*-syn expression in ipsilateral SNc was detected with the anti-h*α*-syn (A53T) antibody. The results showed that mCherry-positive cells can be observed, wherein the mCherry and TH colabeled cells were approximately 85% of the total mCherry-positive cells (Figures 1(a) and 1(b)) at 15 days after viral injection. These mCherry-positive cells gradually increased from 15 days to 3 months and 7 months after viral injection. The number of positive cells was higher in 7 months after viral injection than that after 3 months (Figures 1(c) and 1(d)). At 3 months after viral injection, h*α*-syn expression significantly increased in the SNc injected with AAV9-synapsin-mCherry-human SNCA (A53T) compared with that of animals injected with AAV9-synapsin-mCherry (Figures 1(e) and 1(f)). At 7 months after viral injection, h*α*-syn expression increased in the SNc (Figures 1(g) and 1(h)). These results suggest that AAV9-synapsin-mCherry-human SNCA (A53T) continuously operates in the SNc and produces the h*α*-syn (A53T) protein.

### 3.2. Pathological Characteristics of *α*-syn in the SNc

**Figure 2:**
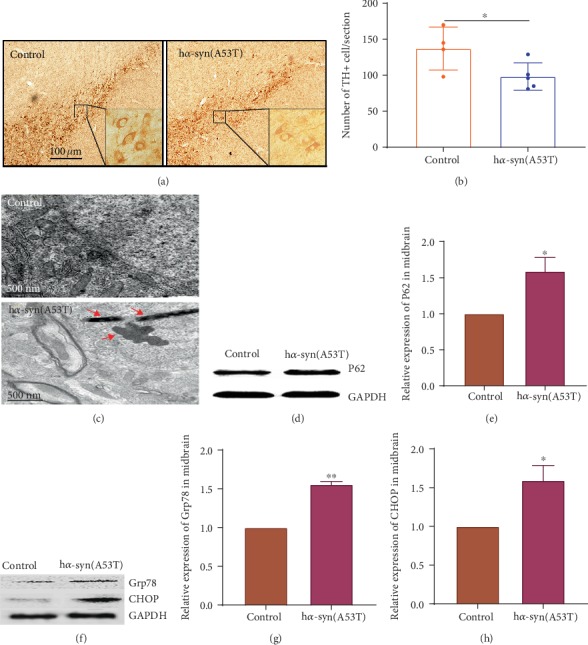
Pathological characteristics of h*α*-syn in the SNc at 7 months after viral injection. (a) Representative images showing TH-positive cells in the SNc. (b) Number of TH-positive cells in the SNc (*n* = 5). (c) Representative images showing the inclusion of body-like structures in the SNc by using a transmission electron microscope (note: the red arrows indicate the inclusion of body-like structures in neurons). (d) p62 expression in ipsilateral midbrain tissues by using Western blot analysis. (e) Quantitative analysis of p62 expression in the ipsilateral midbrain by using image J software (*n* = 4). (f) Grp78 and CHOP expression levels in ipsilateral midbrain tissues by using Western blot analysis. (g, h)09 Quantitative analysis of Grp78 and CHOP expression levels in ipsilateral midbrain using image J software, respectively (*n* = 4). ^∗^*p* < 0.05 and ^∗∗^*p* < 0.01 compared with the control group.

At 7 months after the injection of AAV9-synapsin-mCherry-human SNCA (A53T) in the right SNc, TH-positive cells in the SNc decreased by approximately 35% (Figures 2(a) and 2(b)) compared with the control group. SNc neurons were observed under an electron microscope, and the inclusion of body-like structures (Figure 2(c)) was observed in the h*α*-syn group. p62, an adaptor protein which regulated the autophagy, was detected using WB, and the results showed that the expression level of p62 was significantly higher in the *α*-syn group than that in the control group (Figures 2(d) and 2(e)). The expression levels of Grp78 and CHOP were detected, and the results showed that the expression levels of Grp78 and CHOP protein were significantly higher in the h*α*-syn group than those in the control group (Figures 2(f)–2(h)). These results suggest that the high expression of h*α*-syn in the SNc leads to abnormal lysosome degradation, endoplasmic reticulum stress, and dopamine neuron loss.

### 3.3. Effect of h*α*-syn on the Ipsilateral Striatum

**Figure 3:**
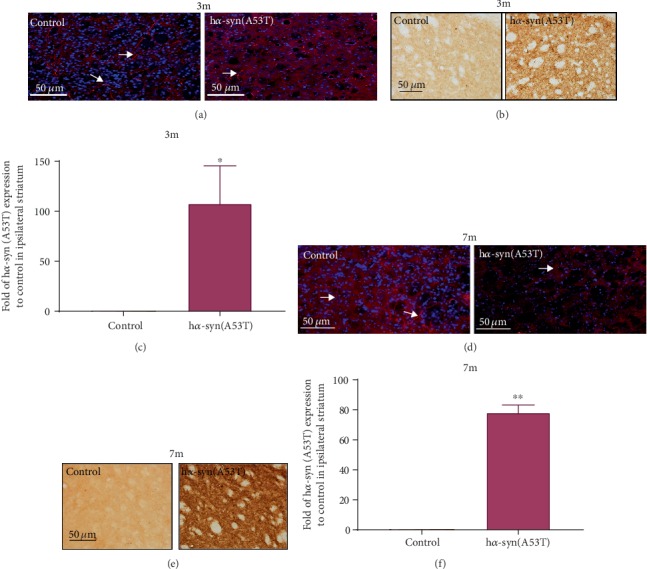
High h*α*-syn expression in the ipsilateral striatum. (a) Representative images showing mCherry-positive nerve fibers in the ipsilateral striatum at 3 months after viral injection. (b) Representative images showing h*α*-syn expression in the ipsilateral striatum by using immunohistochemistry at 3 months after viral injection. (c) Quantitative analysis of h*α*-syn expression in the ipsilateral striatum at 3 months after viral injection (*n* = 3). (d) Representative images showing mCherry-positive nerve fibers in the ipsilateral striatum at 7 months after viral injection. (e) Representative images showing h*α*-syn expression in the ipsilateral striatum by using immunohistochemistry at 7 months after viral injection. (f) Quantitative analysis of h*α*-syn expression in the ipsilateral striatum at 7 months after viral injection. (*n* = 5). ^∗^*p* < 0.05 and ^∗∗^*p* < 0.01 compared with the control group.

At 3 months after the injection of AAV9-synapsin-mCherry-human SNCA (A53T) or AAV9-synapsin-mcherry, mCherry-positive fibers (Figure 3(a)) were found in the ipsilateral striatum. h*α*-syn expression in the ipsilateral striatum was detected by using the anti-h*α*-syn antibody. The expression level of h*α*-syn was significantly higher than that of the control group (Figures 3(b) and 3(c)). At 7 months after injection, mCherry-positive nerve fibers (Figure 3(d)) were found in the striatum, and h*α*-syn expression was significantly higher than that in the control group (Figures 3(e) and 3(f)). These results suggest that the neurons with high h*α*-syn expression in the SNc can project in the striatum, resulting in the increase of h*α*-syn expression in the striatum and the formation of *α*-syn pathology.

### 3.4. Effects of h*α*-syn on the Synaptic Structure and Function in the Striatum

**Figure 4:**
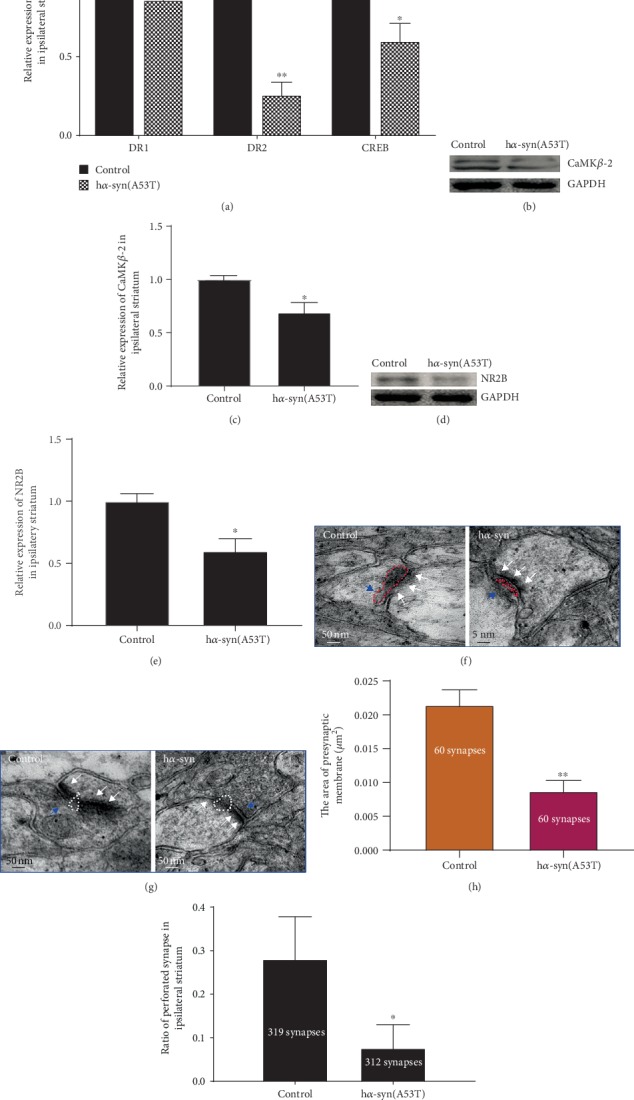
Changes in the synaptic structure and function at 7 months after viral injection. (a) mRNA levels of DR1, DR2, and CREB in the ipsilateral striatum (*n* = 3). (b) CaMK*β*-2 expression in the ipsilateral striatum by using Western blot analysis. (c) Quantitative analysis of CaMK*β*-2 expression in the ipsilateral striatum (*n* = 4). (d) NR2B expression in the ipsilateral striatum by using Western blot analysis. (e) Quantitative analysis of NR2B expression in the ipsilateral striatum (*n* = 4). (f, g) Representative images showing the synaptic ultrastructure in the dorsal ipsilateral striatum by using the transmission electron microscope (note: the red dashed line indicates the synaptic vesicle, white dashed line indicates the perforated synapse, white arrows indicate the postsynaptic membrane, and blue arrows indicate the presynaptic membrane area). (h) Mean area of the presynaptic membrane in 60 synapses (*n* = 3). (i) Ratio of the perforated synapse in more than 300 synapses (*n* = 3). ^∗^*p* < 0.05 and ^∗∗^*p* < 0.01 compared with the control group.

At 7 months after the injection of AAV9-synapsin-mCherry-human SNCA (A53T) or AAV9-synapsin-mCherry, the changes of synaptic plasticity-related molecules and synaptic structure in the ipsilateral striatum were detected. The results showed that the levels of D2R and CREB mRNA decreased (Figure 4(a)) and the expression levels of CaMK*β*-2 and NR2B decreased (Figures 4(b)–4(e)) in the h*α*-syn group compared with those of the control group. The changes in the synaptic structure of the striatum were observed and measured by using electron microscopy, and the presynaptic membrane area was measured in 60 asymmetric synapses per group, and the results showed that the presynaptic membrane area in the h*α*-syn group was smaller than that in the control group (Figures 4(f) and 4(h)). The proportion of perforated synapses was calculated, and the results showed that the proportions of perforated synapses were approximately 7% and 28% in the h*α*-syn and control groups, respectively (Figures 4(g) and 4(i)). These results suggest that the *α*-syn pathology in the striatum leads to the decrease of synaptic plasticity and activity in the striatum.

### 3.5. Changes of Motor Behavior

**Figure 5:**
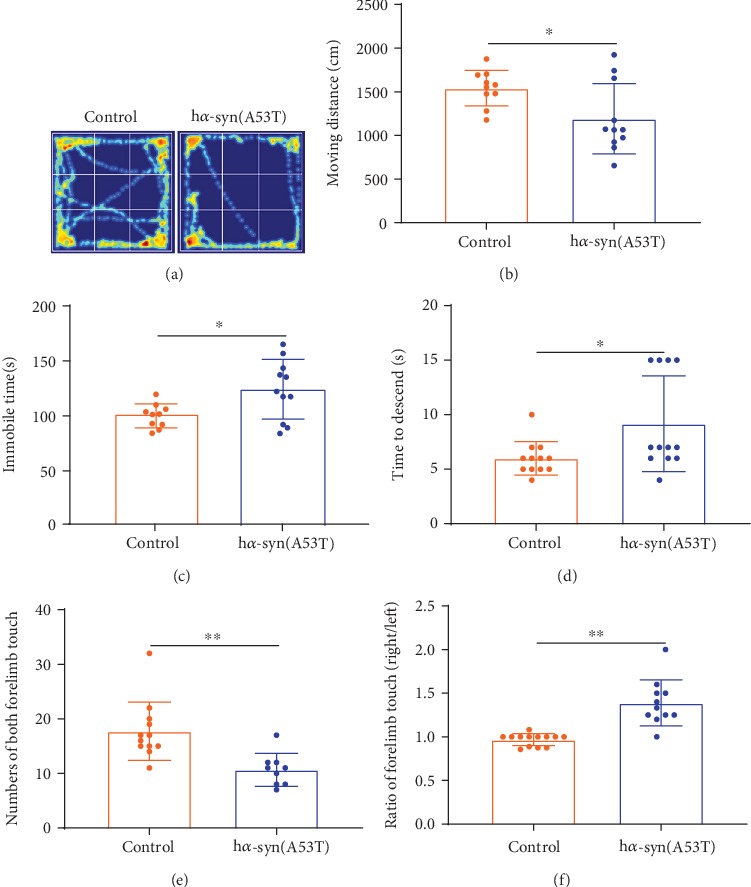
Changes in motor behavior at 7 months after viral injection. (a) Representative images showing the movement track in the open field test. (b) Moving distance within 5 min. (c) Immobile time within 5 min. (d) Time spent to descend from the top of the pole. (e) Number of times both forelimbs touched the cylinder wall within 10 min. (f) Proportion of the right and left forelimbs touching the cylinder wall within 10 minutes. *n* = 15, ^∗^*p* < 0.05, and ^∗∗^*p* < 0.01 compared with the control group.

Motor behavior was detected in the mice injected with AAV9-synapsin-mCherry-human SNCA (A53T) or AAV9-synapsin-mCherry at 7 months after injection. The spontaneous activity in mice was measured by using OFT, and the results showed that the movement distance decreased and immobile time increased (Figures 5(a)–5(c)) in the h*α*-syn group compared with those of the control group. Motor capability was measured by using the pole descent test, and the results showed that the time spent in the h*α*-syn group was longer than that in the control group (Figure 5(d)). The coordination capability of forelimbs was measured by using the cylinder test, and the results showed that the number of times the animal performed forelimb lifting significantly decreased (Figure 5(e)), and the animal-executed right forelimb touch times were significantly more than that when it accomplished left forelimb touch times (Figure 5(f)) in the h*α*-syn group compared with those of the control group. These results suggest that the h*α*-syn expression (A53T) in unilateral SNc caused mild motor dysfunction in mice after 7 months.

### 3.6. Effect of h*α*-syn on the Olfactory Bulb

**Figure 6:**
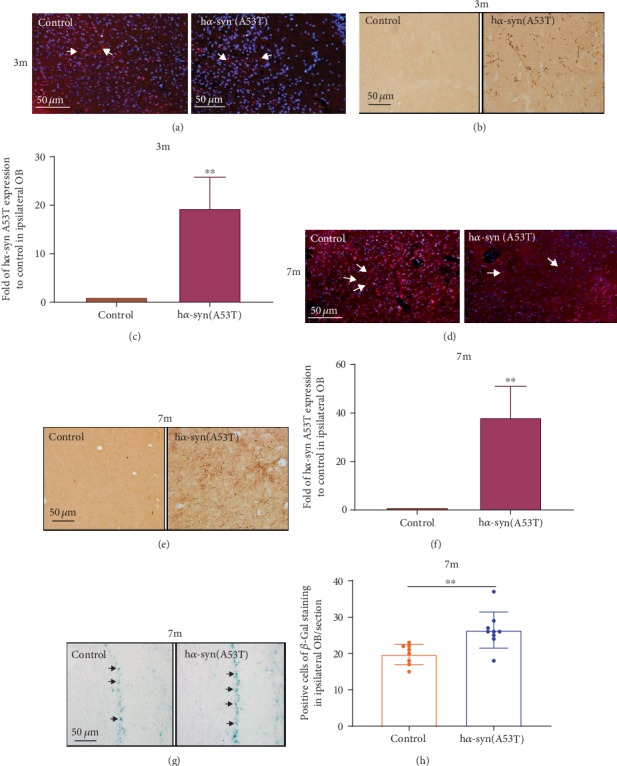
High h*α*-syn expression in the ipsilateral olfactory bulb. (a) Representative images showing mCherry-positive nerve fibers in the ipsilateral olfactory bulb at 3 months after viral injection. (b) Representative images showing h*α*-syn expression in the ipsilateral olfactory bulb by using immunohistochemistry at 3 months after viral injection. (c) Quantitative analysis of h*α*-syn expression in the ipsilateral olfactory bulb at 3 months after viral injection (*n* = 3). (d) Representative images showing mCherry-positive nerve fibers in the ipsilateral olfactory bulb at 7 months after viral injection. (e) Representative images showing h*α*-syn expression in the ipsilateral olfactory bulb by using immunohistochemistry at 7 months after viral injection. (f) Quantitative analysis of h*α*-syn expression in the ipsilateral olfactory bulb at 7 months after viral injection (*n* = 5). (g) Representative images showing positive *β*-gal staining in the olfactory bulb at 7 months after viral injection. (h) Number of *β*-gal-positive cells in the olfactory bulb at 7 months after viral injection (*n* = 5). ^∗∗^*p* < 0.01 compared with the control group.

At 3 months after the injection of AAV9-synapsin-mCherry-human SNCA (A53T) or AAV9-synapsin-mCherry, mCherry-positive nerve fibers (Figure 6(a)) were found in the ipsilateral olfactory bulb. h*α*-syn expression in the ipsilateral olfactory bulb was detected by anti-h*α*-syn antibody, and the results showed that h*α*-syn expression was significantly higher than that in the control group (Figures 6(b) and 6(c)). Seven months after viral injection, mCherry-positive nerve fibers (Figure 6(d)) were found in the ipsilateral olfactory bulb, and h*α*-syn expression was significantly higher than that in the control group (Figures 6(e) and 6(f)). Aging in olfactory bulb cells was detected via *β*-gal staining, and the results showed that the number of *β*-gal-positive cells located in the mitral cell layer of the olfactory bulb was significantly higher in the h*α*-syn group than that in the control group (Figures 6(g) and 6(h)). These results suggest that high h*α*-syn expression accelerates mitral cell aging in the olfactory bulb.

### 3.7. Changes in the Olfactory Function

**Figure 7:**
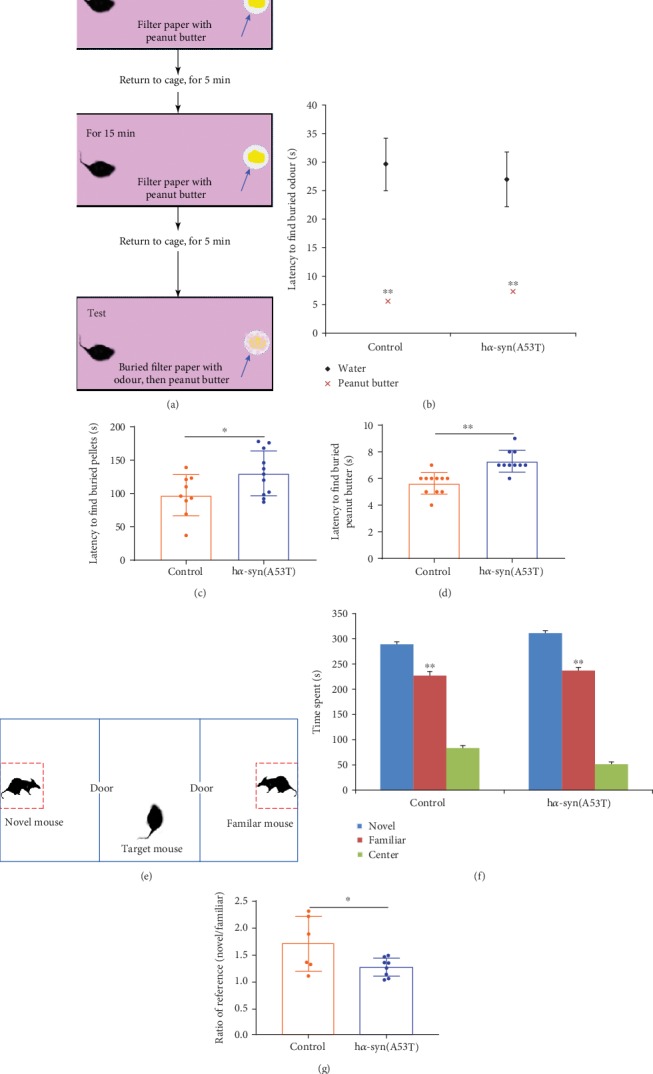
Changes in olfactory function at 7 months after viral injection. (a) Schematic of olfactory sensitivity test. (b) Latency to find the buried odor. (c) Latency to find the buried food. (d) Latency to find the buried peanut butter. (e) Schematic of the three-chamber social test. (f) Time spent in the novel mice resident, familiar mice resident, and central chambers. (g) Proportion of the time spent in the novel mice resident chamber and the time spent in familiar mice resident chamber within 10 min. ^∗^*p* < 0.05 and ^∗∗^*p* < 0.01 compared with the control group, *n* = 15.

At 7 months after the injection of AAV9-synapsin-mCherry-human SNCA (A53T) or AAV9-synapsin-mCherry, the ability to recognize odor was evaluated. The results showed that the length of time until the mice found the peanut butter was significantly shorter than that until they found water in the two groups (Figures 7(a) and 7(b)). This result showed that the odor-based exploration ability of the two groups of mice was normal. In the food burial experiment, the length of time until the mice found all the food chow was longer in the h*α*-syn group than that in the control group (Figure 7(c)). Based on the order of memory training, the odor memory ability was detected, and the results showed that the length of time until the mice found familiar odors was longer in the h*α*-syn group compared with that of the control group (Figures 7(a) and 7(d)). Furthermore, the social behavior on the basis of olfactory recognition was detected, and the results showed that the length of time spent by the unfamiliar conspecific mouse was longer than that of the familiar conspecific mouse in both groups (Figures 7(e) and 7(f)), whereas the length of time spent of the unfamiliar mouse was shorter in the h*α*-syn group than that in the control group (Figure 7(g)).

## 4. Discussion

The study of *α*-syn has become an important aspect in PD research when the involvement of SNCA (encoding *α*-syn protein) gene mutation was first reported in the pathogenesis of familial hereditary PD in 1997 [38]. A transgenic animal model has since been established. Although the study based on this animal model has deepened the understanding of PD pathology, the function of *α*-syn, especially the mutated *α*-syn in the pathological formation of PD, remains unclear [39–41]. The high *α*-syn expression or the outcome of PD in the brain cannot be confirmed as the cause of PD. Our study found that the h*α*-syn expression (A53T) in the SNc increased at 3 and 7 months after the unilateral injection of AAV9-synapsin-mCherry-human SNCA (A53T) in the SNc. At 7 months after the injection, the number of dopaminergic neurons in the SNc decreased possibly due to the intracellular protein aggregation and endoplasmic reticulum stress caused by h*α*-syn (A53T). Furthermore, endoplasmic reticulum stress-dependent apoptosis was activated. The h*α*-syn (A53T) generated from SNc neurons that was transported into the striatum along the projecting fibers affected the synaptic structure and efficiency in the striatum, thereby indicating that this phenomenon may be the key mechanism of motor dysfunction. The h*α*-syn (A53T) generated from SNc neurons that was transported into the olfactory bulb along the projecting fibers accelerated cell aging in the mitral layer of the olfactory bulb, possibly contributing to the decrease in olfactory sensitivity.

The persistent high expression of h*α*-syn (A53T) in the SNc, characterized by endoplasmic reticulum stress, protein aggregation, abnormal autophagy, and induction of endoplasmic reticulum-dependent apoptosis, leads to the pathological formation of *α*-syn in the SNc. This condition may cause the loss of dopaminergic neurons in the SNc [42–44]. The cerebral regional specificity of PD pathology has constantly attracted considerable attention from researchers. Studies in different animal models have shown that the damage of dopaminergic neurons in the SNc is clear. This condition may be due to the following reasons: First, many synaptic connections exist in dopaminergic neurons in the SNc [45]. Thus, the frequency of neuron activity is high and accompanied by high metabolism. In addition, dopaminergic neurons in the midbrain express high monoamine oxidase, which can produce many oxygen-free radicals that cause neuron damage [46]. In this study, we excluded the effect of other brain regions on the pathological formation of the SNc by only infecting SNc neurons, which is important in determining the effect of independent expression of *α*-syn in the SNc on the pathological formation of PD.

Although PD pathology involves the basal ganglia in transgenic animal models, whether the pathological formation of striatum causes the damage of dopaminergic neurons in the SNc cannot be confirmed because striatal neurons also express h*α*-syn (A53T) in h*α*-syn transgenic mice. Our study confirms the effects of h*α*-syn (A53T) originating from the SNc on the striatum by using directional viral injections in unilateral SNc. At 3 and 7 months after injection, *α*-syn was expressed in the nerve fibers of the striatum. mCherry labeling confirmed that these fibers originated from the SNc. These results suggest that the h*α*-syn (A53T) in the striatum derived from SNc neurons along its projection fibers affected the neural connection in the striatum. Furthermore, the electron microscopy results showed that the synaptic structure changes involved in the presynaptic membrane area and the ratio of perforated synapse in the dorsal striatum decreased, suggesting the active synapses reduced.

Olfactory dysfunction is significant for nonmotor symptoms of PD. However, whether olfactory dysfunction is caused by the pathology of SNc neurons remains unclear. In this study, h*α*-syn (A53T) was found in the nerve fibers of the ipsilateral olfactory bulb after viral injections in unilateral SNc. In addition, mCherry labeling confirmed that these nerve fibers directly originated from the SNc, thereby indicating that the pathogenesis of dopaminergic neurons in the SNc affected the olfactory bulb through its projection fibers and accelerated the cell aging in the mitral cell layer of the olfactory bulb, subsequently causing the decrease in olfactory sensitivity.

The nerve fibers projected into the striatum and the olfactory bulb may be the direct cause of the changes in the motor function and olfactory dysfunction in PD, respectively. Neurons with h*α*-syn (A53T) expression in the local brain region affect the function of distant cerebral regions by projecting fibers. The pathological deposition of h*α*-syn affects not only synaptic structure, synaptic plasticity, and synaptic efficiency but also accelerates neural aging in distant cerebral regions, thereby indicating that this phenomenon may be an important pathological feature of h*α*-syn (A53T).

Previous studies have shown that *α*-syn can spread across brain regions [47–49]. Our study showed that h*α*-syn (A53T) expressed in SNc neurons can arrive at distant cerebral regions along projecting fibers. Most studies believe that AAV only exists in infected neurons, suggesting that the majority of the h*α*-syn (A53T) in distant brain regions is directly spread along the projecting fibers [50]. However, studies have suggested that AAV demonstrates a transsynaptic effect, indicating that some h*α*-syn (A53T) may spread to distant brain regions in more than two levels of neurons [51]. Therefore, the primary means of *α*-syn transmission is along the nerve fibers from one region to another.

There are many types of SNCA mutations, and the highest frequency is A53T, A30P, and E46K. The *α*-syn protein encoded by A53T mutation is easy to form *α*-helix structure, which leads to fibrosis and accumulation, resulting in dysfunction of degradation depending on the autophagy-lysosomal pathway [52]. Consistently, the expression of h*α*-syn in striatum and olfactory bulb increased with time in our experiment. However, it is difficult to distinguish the conformation of *α*-syn in vivo.

## 5. Conclusion

The directional injection of AAV9-synapsin-mCherry-human SNCA (A53T) in the SNc can partially induce PD-like pathology and symptoms. The high expression of h*α*-syn (A53T) in SNc not only leads to the pathogenesis in the SNc but also decreases the synaptic activity in the striatum and accelerates cell aging in the olfactory bulb through the projection of fibers from the SNc, thereby causing motor and nonmotor dysfunctions in the development of PD pathogenesis.

## Data Availability

The data used to support the findings of this study are available from the corresponding author upon request.

## Supplementary Materials

Supplementary Materials We detected the expression of *γ*-H2X in olfactory bulb by immunohistochemical technique, and the level of positive cells in mitral cells was similar with that of *β*-gal staining. The results showed that the number of *γ*-H2X-positive cells located in the mitral cell layer of the olfactory bulb was significantly higher in the h*α*-syn group than that in the control group (Figure S1 a and b). Figure S1: (a) representative images showing positive *γ*-H2X in the olfactory bulb at 7 months after viral injection. (b) Number of *γ*-H2X-positive cells in the olfactory bulb at 7 months after viral injection. ^∗∗^*p* < 0.01 compared with the control group, *n* = 5. We have provided all original pictures of Western blot. Figure S2: the original pictures of Western blot. (A) indicates control group and (B) indicates h*α*-syn(A53T) group. (a) The expression of p62 in midbrain. (b) The expression of Grp78 and CHOP in midbrain. (c) The expression of Grp78 in midbrain. (d) The expression of CHOP in midbrain. (e) The expression of CaMK*β*-2 in ipsilateral striatum. (f) The expression of GAPDH in ipsilateral striatum. (g) The expression of CaMK*β*-2 in ipsilateral striatum. (h) The expression of NR2B in ipsilateral striatum.
