# Supplementary material for: Noncanonical Roles of hα-syn (A53T) in the Pathogenesis of Parkinson's Disease: Synaptic Pathology and Neuronal Aging
Source: Neural Plast. 2020 Mar 21;2020:6283754. doi: 10.1155/2020/6283754 (PMC7115172; doi:10.1155/2020/6283754)
Supplement: Supplementary Materials — We detected the expression of γ-H2X in olfactory bulb by immunohistochemical technique, and the level of positive cells in mitral cells was similar with that of β-gal staining. The results showed that the number of γ-H2X-positive cells located in the mitral cell layer of the olfactory bulb was significantly higher in the hα-syn group than that in the control group (Figure S1 a and b). Figure S1: (a) representative images showing positive γ-H2X in the olfactory bulb at 7 months after viral injection. (b) Number of γ-H2X-positive cells in the olfactory bulb at 7 months after viral injection. ∗∗p < 0.01 compared with the control group, n = 5. We have provided all original pictures of Western blot. Figure S2: the original pictures of Western blot. (A) indicates control group and (B) indicates hα-syn(A53T) group. (a) The expression of p62 in midbrain. (b) The expression of Grp78 and CHOP in midbrain. (c) The expression of Grp78 in midbrain. (d) The expression of CHOP in midbrain. (e) The expression of CaMKβ-2 in ipsilateral striatum. (f) The expression of GAPDH in ipsilateral striatum. (g) The expression of CaMKβ-2 in ipsilateral striatum. (h) The expression of NR2B in ipsilateral striatum. [file 6283754.f1.zip › fig S2.pdf]

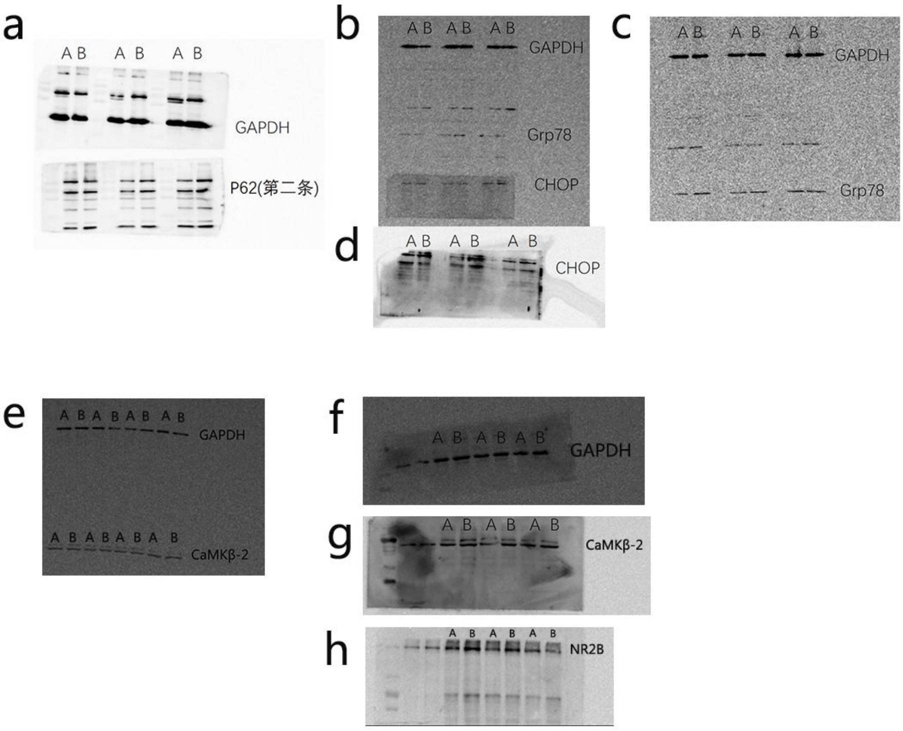

Figure S2.The original pictures of WB.

(a) p62

(b) Grp78 and CHOP

(c) Grp78

(d) CHOP

(e) CaMK β -2

(f) GAPDH

(g) CaMK β -2

(h) NR2B

NOTE:A=control group, B=h α -syn(A53T) group
